# THE ROLE OF SOCIAL MEDIA NETWORKS FOR AGING ADULTS: PANACEA, PLAGUE, OR BEEN THERE, DONE THAT?

**DOI:** 10.1093/geroni/igz038.767

**Published:** 2019-11-08

**Authors:** Neil H Charness

**Affiliations:** Florida State University, Tallahassee, Florida, United States

## Abstract

Billions of people around the world rely on (or are addicted to) technology-based social networks such as Facebook. At first glance, particularly for aging adults with mobility challenges, internet-based networking seems like a panacea. At second glance, navigating through the thicket of ‘bots’, fraudsters and “fake friends” may turn out to be a plague. At third glance, technology-based interaction platforms are not that new (telegraph, telephone) and not that unusual. I examine the population-level trends in social network use by aging adults and discuss a recent CREATE intervention study, PRISM, that used a computer-based platform to try to reduce social isolation and loneliness in older adults at risk for social isolation.

